# Supplementary material for: Uncovering the endogenous features of potassium salts’ global transfer: A complex network perspective
Source: PLoS One. 2024 Mar 6;19(3):e0295139. doi: 10.1371/journal.pone.0295139 (PMC10917254; doi:10.1371/journal.pone.0295139)
Supplement: S1 Table — (DOCX) [file pone.0295139.s001.docx]

**S1 Table. Node centrality of top 10 countries from 2000 to 2021**

| Closeness centrality | | | | | | | | | |  |
| --- | --- | --- | --- | --- | --- | --- | --- | --- | --- | --- |
| Year | Rank | | | | | | | | | |
|  | 1 | 2 | 3 | 4 | 5 | 6 | 7 | 8 | 9 | 10 |
| 2000 | Germany | Argentina | Ecuador | USA | United Kingdom | Israel | Russian Federation | France | Mozambique | Canada |
| 2001 | South Africa | Germany | Ecuador | United Arab Emirates | Mozambique | Dominican Rep. | Panama | USA | Costa Rica | Guatemala |
| 2002 | Germany | Argentina | Panama | USA | Brazil | United Kingdom | Russian Federation | Israel | Costa Rica | France |
| 2003 | Germany | Costa Rica | USA | Guatemala | Russian Federation | United Kingdom | Israel | Netherlands | Canada | France |
| 2004 | Germany | USA | Israel | Costa Rica | Russian Federation | United Kingdom | Canada | Belgium | Jordan | Netherlands |
| 2005 | Germany | USA | United Kingdom | Israel | Russian Federation | Netherlands | Canada | Jordan | China | Belgium |
| 2006 | South Africa | Argentina | Germany | Costa Rica | Mauritius | USA | United Kingdom | Israel | Russian Federation | China |
| 2007 | Peru | Germany | Guatemala | Colombia | USA | Israel | United Kingdom | Russian Federation | Netherlands | China |
| 2008 | Germany | USA | Israel | United Kingdom | Russian Federation | India | Netherlands | Belgium | China | Jordan |
| 2009 | Germany | Kenya | Uzbekistan | USA | Israel | United Kingdom | India | Russian Federation | Belgium | Netherlands |
| 2010 | Germany | China, Hong Kong SAR | USA | Russian Federation | Israel | United Kingdom | Belgium | Kenya | Belarus | Canada |
| 2011 | Germany | Senegal | Mali | Burkina Faso | USA | Israel | Russian Federation | United Kingdom | Belarus | Spain |
| 2012 | South Africa | Germany | Dominican Rep. | USA | United Kingdom | Russian Federation | Israel | Belarus | Netherlands | Spain |
| 2013 | South Africa | Germany | Cote d'Ivoire | Guatemala | USA | Saudi Arabia | Nicaragua | Ecuador | Honduras | Russian Federation |
| 2014 | South Africa | Germany | Russian Federation | Free Zones | El Salvador | USA | Belarus | China | Israel | United Kingdom |
| 2015 | Germany | Russian Federation | Saint-Barthélemy | USA | Belarus | China | United Kingdom | Israel | Spain | Chile |
| 2016 | South Africa | Nigeria | Germany | Belarus | USA | Suriname | Russian Federation | China | United Kingdom | Israel |
| 2017 | Morocco | Costa Rica | Kenya | Germany | USA | Saint-Barthélemy | Pakistan | Iceland | Belarus | China |
| 2018 | Mozambique | United Rep. of Tanzania | Togo | Honduras | Lebanon | Cote d'Ivoire | Holy See (Vatican City State) | Cameroon | Angola | Tunisia |
| 2019 | Germany | Cameroon | China, Hong Kong SAR | China | Belarus | Russian Federation | USA | Montenegro | Israel | United Kingdom |
| 2020 | Germany | Russian Federation | Belarus | China | Panama | United Rep. of Tanzania | Equatorial Guinea | USA | United Kingdom | Czechia |
| 2021 | South Africa | Germany | Qatar | Belarus | China | Russian Federation | Namibia | USA | Israel | Czechia |
| Betweenness centrality | | | | | | | | | | |
| Year | Rank | | | | | | | | | |
|  | 1 | 2 | 3 | 4 | 5 | 6 | 7 | 8 | 9 | 10 |
| 2000 | Germany | France | USA | United Kingdom | India | Switzerland | Spain | Malaysia | Israel | Brazil |
| 2001 | USA | France | Germany | China | India | Australia | China, Hong Kong SAR | Malaysia | Philippines | United Kingdom |
| 2002 | Russian Federation | Germany | India | USA | France | China | Netherlands | Chile | United Kingdom | South Africa |
| 2003 | Germany | Canada | Spain | Singapore | USA | Russian Federation | India | France | South Africa | Belgium |
| 2004 | Germany | Russian Federation | China | Canada | India | USA | France | Brazil | Belgium | United Kingdom |
| 2005 | USA | United Kingdom | France | Australia | Russian Federation | Germany | China | Chile | India | Belgium |
| 2006 | USA | Germany | Australia | Canada | Russian Federation | France | Singapore | China | Czechia | Belgium |
| 2007 | USA | Russian Federation | Germany | China | Belgium | India | France | Australia | Canada | Poland |
| 2008 | USA | Germany | France | Australia | United Kingdom | China | Belgium | Czechia | South Africa | India |
| 2009 | Chile | India | France | Peru | Colombia | United Kingdom | Germany | USA | Russian Federation | Venezuela |
| 2010 | Austria | Dominican Rep. | Japan | United Kingdom | Colombia | Czechia | Latvia | Sudan (...2011) | Ecuador | Panama |
| 2011 | Germany | India | Chile | China | USA | France | Russian Federation | Argentina | Australia | Denmark |
| 2012 | Germany | India | USA | Russian Federation | France | Singapore | China | United Kingdom | Spain | Austria |
| 2013 | Germany | Russian Federation | Australia | France | USA | Malaysia | Canada | Belgium | India | China |
| 2014 | Russian Federation | Canada | USA | China | Germany | India | United Kingdom | Australia | Indonesia | Belgium |
| 2015 | Netherlands | Germany | USA | France | Russian Federation | Canada | Spain | China | United Kingdom | Belgium |
| 2016 | Germany | Netherlands | China | Canada | USA | France | India | Russian Federation | Chile | Malaysia |
| 2017 | Germany | USA | Netherlands | India | Russian Federation | Canada | Czechia | France | Japan | China |
| 2018 | Germany | Netherlands | Canada | France | Russian Federation | USA | Spain | India | China | Chile |
| 2019 | China | Netherlands | Germany | Canada | USA | Malaysia | Russian Federation | France | Nicaragua | India |
| 2020 | Netherlands | China | Russian Federation | USA | Germany | Czechia | Spain | France | India | Hungary |
| 2021 | Germany | USA | Russian Federation | Netherlands | Spain | Brazil | Bolivia | Chile | Canada | China |
|  |  |  |  |  |  |  |  |  |  |  |
