# Supplementary material for: Uncovering the endogenous features of potassium salts’ global transfer: A complex network perspective
Source: PLoS One. 2024 Mar 6;19(3):e0295139. doi: 10.1371/journal.pone.0295139 (PMC10917254; doi:10.1371/journal.pone.0295139)
Supplement: Attachment — Submitted filename: Response to Reviewers.docx [file pone.0295139.s002.docx]

Dear editor and reviewer:

Thank you for your comments concerning our manuscript entitled “Uncovering the endogenous features of the potassium salts’ global transfer: a complex network perspective”. Those comments are really valuable and helpful for revising and improving our paper. We have studied the comments carefully and revised our paper according to your advices. We responded to your comments point by point as follows:

**Editor #1:** Manuscript Number: PONE-D-23-16699

Title: Uncovering the endogenous features of the potassium salts’ global transfer: a complex network perspective

Journal: Plos One

Journal requirements:

[Answer] Thank you for your suggestion. We have revised the manuscript format according to the journal's requirements. Besides, we have uploaded the related files when resubmitting the manuscript.

**Reviewer #1：**Manuscript Number: PONE-D-23-16699

Title: Uncovering the endogenous features of the potassium salts’ global transfer: a complex network perspective

Journal: Plos One

Comment 1: Coming from an economic perspective, it is perhaps not surprising that the authors have not cited some of the recent (but pre-dating the war in Ukraine) publications on this topic, written from the perspective of mineral resources and agriculture. Recent reviews and analyses include:

[Answer] Thank you for your suggestion. We have carefully read these papers, which is very helpful for improving our manuscript. Since this manuscript focuses on the risk of trade networks, we did not cite all of them and selected two.

Comment 2: The analysis that has been carried out is clearly explained, but I think that non-specialists may need more help in interpreting the text and the figures. For example, in Figure 3, maybe the major exporting countries could be named in bold or upper case text, so they are clearly visible (the arrows that cross the diagram are necessarily broad, so at first it is difficult to see the direction). Again, in Figure 4 the arrowheads are hard to see as they are at the end of the arrow; it would help if arrowheads were drawn also in the middle of the arrow, to emphasise the flow. Figure 4 could be clarified if the three categories were briefly summarised in the caption or indeed with additional text in the figure. I appreciate this is done in the main text. However, the groups are there described in terms of groups of countries, without explaining simply, ie in a very few words, why they are grouped as they are. This would help readers from other disciplinary backgrounds.

[Answer] Thank you for your suggestion. We have adjusted Fig.3 and Fig.4 and made the font size of the major exporting countries in Fig.3 larger. Moreover, in Fig.4, we have removed the arrow of trade relations and determined the direction of trade by the color of the line. In addition, we explain how to determine the group in a few words. The revisions we made are as follows:

“As per Eq. (7), this paper quantifies the modularity of countries in the PTN and subsequently categorizes communities according to their modularity.”

Comment 3: Figure 4 is especially interesting. Although some African countries are coloured, there are no arrows showing where their imports come from. Why is this? Could you comment on why Africa is not part of global trade in potassium salts?

[Answer] Thank you for your suggestion. This misunderstanding occurred because we did not explain it clearly. In Fig.4, the same color indicates the same community. Trade flows only include major trade countries. The figure will look very messy if we put all the trade flows. Therefore, we screen out trade relationships with relatively small trade volumes.

Comment 4: An additional topic that could be considered in the Discussion is the possibility for disruption of the market by new products. The example I have in mind is polyhalite, and the Anglo American Woodsmith Mine (https://uk.angloamerican.com/the-woodsmith-project) that is designed to produce 20 million tonnes of polyhalite, an alternative to conventional potassium salts, for sale into the global fertilizer market. This will compete with conventional sources, and could have a major impact on trade patterns. However, although there is some trade in this material from an existing mine (ICL Boulby), it is very small. I believe, however, that China has large deposits of polyhalite that are being considered for exploitation, or are being mined at present. It would be good if the authors could comment on these new materials, although of course a similar analysis of their trade is not yet possible.

[Answer] Thank you for this essential information. After our discussion, we prefer to focus on the trade issue of potassium salts. For polyhalite, this may be covered in future research. However, we have added a small discussion about polyhalite to arouse readers' thinking. The revisions we made are as follows:

“Notably, the discovery and development of potassium deposits in Canada have changed the global supply pattern. The UK's plans for large-scale polyhalite mining will provide an additional source of potassium, which may once again change the global supply pattern.”

Comment 5: Minor comments relate mainly to the few occasions where correction to the excellent written English would improve the manuscript.

[Answer] Thank you for your suggestion. These minor comments are essential for improving the manuscript. We revised the manuscript based on these comments.

In addition, we tried our best to improve the manuscript and made some changes in the manuscript. These changes will not influence the content and framework of the paper. And here we did not list the other changes.

We appreciate for Editors/Reviewers’ warm work earnestly, and hope that the correction will meet with approval.

Once again, thank you very much for your comments and suggestions.
